# Proximate Analyses and Amino Acid Composition of Selected Wild Indigenous Fruits of Southern Africa

**DOI:** 10.3390/plants10040721

**Published:** 2021-04-08

**Authors:** Nozipho P. Sibiya, Eugenie Kayitesi, Annah N. Moteetee

**Affiliations:** 1 Department of Botany and Plant Biotechnology, APK Campus, University of Johannesburg, P.O. Box 524, Auckland Park, Johannesburg 2006, South Africa; npsibiyazulu@gmail.com; 2 Department of Biotechnology and Food Technology, DFC Campus, University of Johannesburg, P.O. Box 17011, Doornfontein, Johannesburg 2028, South Africa; eugenie.kayitesi@up.ac.za; 3 Department of Consumer and Food Sciences, University of Pretoria, Pretoria 0028, South Africa

**Keywords:** indigenous fruits, mineral, nutrition, proximate, Southern Africa, vitamin

## Abstract

A literature survey revealed that several wild indigenous Southern African fruits had previously not been evaluated for their proximate and amino acid composition, as well as the total energy value (caloric value). Fourteen species including *Carissa macrocarpa*, *Carpobrotus edulis*, *Dovyalis caffra*, *Halleria lucida*, *Manilkara mochisia*, *Pappea capensis*, *Phoenix reclinata*, and *Syzygium guineense* were analyzed in this study. The nutritional values for several species such as *C. edulis*, *H. lucida*, *P. reclinata*, and *M. mochisia* are being reported here for the first time. The following fruits had the highest proximate values: *C. macrocarpa* (ash at 20.42 mg/100 g), *S. guineense* (fat at 7.75 mg/100 g), *P. reclinata* (fiber at 29.89 mg/100 g), and *H. lucida* (protein at 6.98 mg/100 g and carbohydrates at 36.98 mg/100 g). Essential amino acids such as histidine, isoleucine, lysine, methionine, phenylalanine, tryptophan, and valine were reported in all studied indigenous fruits. The high protein content in *H. lucida* was exhibited by the highest amino acid quantities for histidine. However, the fruits are a poor source of proteins since the content is lower than the recommended daily intake. The jacket-plum (*Pappea capensis*), on the other hand, meets and exceeds the required daily intake of lysine (0.0003 g/100 g or 13 mg/kg) recommended by the World Health Organization.

## 1. Introduction

The Food Agricultural Organisation [1] estimates that only 10,000 of the 300,000 known plant species have been used for human food since the origin of agriculture. Only about 150–200 of these have been commercially cultivated, with only rice, wheat, maize, and potatoes supplying 50% of the world’s caloric intake. This therefore indicates that many plants with the potential to improve food and nutrition security are not yet mainstreamed [2]. Presently, malnutrition is a major health problem in Africa, for example, the number of nutrition stunted children increased from 50.4 to 58.5 million between the year 2000 to 2016 [3,4]. According to Duguma [5], the use of wild indigenous fruits could combat malnutrition and improve food security. Most foods from the wild play an important part in supplying nutrition to communities during periods of food scarcity as they can be consumed as snacks in emergency demands.

Nutrition assessment is the best way to determine whether or not people’s nutritional needs are being effectively met [6]. Nutrition analysis of wild edible indigenous fruits provides quality, quantity, and evidence-based information for future research, planning, commercialization, and utilization, all together aiming at eradicating hunger, poverty and reducing the burden of malnutrition in Southern Africa. The proximate technique is a system of food analysis that is divided into five constituents, i.e., ash, moisture, proteins, fats, and carbohydrates [7]. Although the proximate procedure does not give complete nutritional information (i.e., minerals, vitamins, and amino acids), it is a low-cost analysis that is used to track deviations from the quality of foods [8]. Several wild fruits are a good source of carbohydrates, proteins, and fats that may be lacking in some human diets [9]. For example, a study conducted to assess several wild edible indigenous African fruits noted that *Azanza garckeana* (F. Hoffm) Exell & Hillc. (Mutchwa), *Parinari curatellifolia* Benth. (mobola plum), *Strychnos spinosa* Lam. (spiny monkey orange), *Trichilia emetica* Vahl (natal mahogany), and *Ximenia caffra* Sond. (sour plum), gave the highest levels of fibre (45.3 g/100 g), carbohydrates (88.2 g/100 g), fat (31.2 g/100 g), protein (17.0 g/100 g), and ash (11.2 g/100 g), respectively [9].

**Figure 1:**
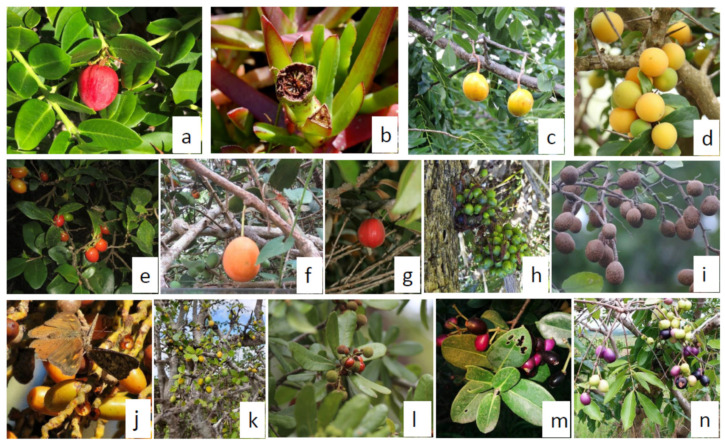
(**a**) *Carissa macrocarpa* (Maryann Robledo; https://www.inaturalist.org/photos/51979217; accessed 6 March 2021), (**b**) *Phoenix reclinata* (Peter Vos; https://www.inaturalist.org/photos/19456167; accessed 6 March 2021), (**c**) *Parinari curatellifolia* (Amadou Bahleman Farid; https://www.inaturalist.org/photos/85363757; accessed 6 March 2021), (**d**) *Garcinia livingstonei* (Magdastlucia, https://www.inaturalist.org/photos/30855552; accessed 6 March 2021), (**e**) *Cordyla africana* (Carl Bodenstaff; https://www.inaturalist.org/photos/18012108; accessed 6 March 2021), (**f**) *Dovyalis caffra* (Sharon Louw; https://www.inaturalist.org/photos/77938897; accessed 6 March 2021), (**g**) *Dovyalis longispina* (Ricky Taylor; https://www.inaturalist.org/photos/28214507; accessed 6 March 2021), (**h**) *Carpobrotus edulis* (Richard Adcock; https://www.inaturalist.org/photos/15319464; accessed 6 March 2021), (**i**) *Syzygium cordatum* (Craig Peter; https://www.inaturalist.org/photos/32006265; accessed 6 March 2021), (**j**) *Syzygium guineense* (Rob Palmer; https://www.inaturalist.org/photos/57946244; accessed 6 March 2021), (**k**) *Pappea capensis* (Ricky Taylor; https://www.inaturalist.org/photos/106750435; accessed 6 March 2021), (**l**) *Englerophytum magalismontanum* (Wolf-Achim and Hanna Roland; https://www.inaturalist.org/photos/15575919; accessed 6 March 2021), (**m**) *Manilkara mochisia* (Shane Ngwenya; https://www.inaturalist.org/photos/112233834; accessed 6 March 2021), (**n**) *Halleria lucida* (Chris Wahlberg; https://www.inaturalist.org/photos/15231592; accessed 6 March 2021).

Amino acids are a combination of two organic substances that combine an amide and an acid and are the building blocks of proteins [10]. Amino acids are essential to the human body to undertake biological processes, which give cells their structure, as well as to transport and store nutrients. There are 20 amino acids that the body cannot survive without; these are divided into essential and non-essential amino acids. Essential amino acids are said to be indispensable, as they cannot be synthesized by the body and can only be obtained from food [11]. This study, therefore, compares the results of the selected wild edible indigenous fruits to the World Health Organization (WHO) recommended daily intake, to see if there are substantial amino acids in fruits. This lays the significance of testing fruits for proteins, among other macronutrients. This study presents the research findings based on the proximate values, amino acids, and energy content of the selected wild edible indigenous fruits of Southern Africa. Some background information (including common names used in South Africa) of the studied indigenous fruits (shown in Figure 1a–m) is summarized as follows:

### 1.1. Carissa macrocarpa (Eckl.) A.DC. (Amathungulu, Big Num-Num)

*Carissa macrocarpa* is a fast-growing thorny shrub or small tree up to 4 m tall. The young branches exude a white milky latex. It has leathery, shiny, dark green leaves that are oval or almost round. *Carissa macrocarpa* is a popular ornamental plant characterized by Y- shaped thorns and oval, large fruits (from which it derives its specific name) that turn red when ripe [12]. There are no known medicinal uses of the fruit however, according to Pfukwa et al. [13], compounds such as Oleanolic acid and B-amyrin from the fruits have been found to have analgesic properties. The fruits are edible raw or cooked and are used to make jams, syrup, pickles, and jelly [14]. Van Wyk [15] rated this plant as having high potential as a new crop.

### 1.2. Carpobrotus edulis (L.) N.E.Br. (Sour Fig, Hottentots Fig; Ghaukum, Ghoenavy, Hottentotsvy, Perdevy, Rankvy, Suurvy, Ikhambi-Lamabulawo, Umgongozi)

*Carpobrotus edulis* is an evergreen, low-growing perennial, which forms dense carpets with branching stems up to 3 m long [16]. The green succulent leaves are triangular in shape and turn reddish with age, the yellow flowers are produced from August-October and later form fleshy indehiscent fruits [17]. *Carpobrotus edulis* is commercialized for its edible fruits, the fruits are also used in traditional medicine to treat various ailments such as earache, hypertension, toothache, tuberculosis, and wounds [13]. As a remedy for constipation, fruits should be eaten followed by drinking brackish water, a syrup made from the fruit is also used for this purpose it is said to have laxative properties. An infusion of the fruits during pregnancy is believed to promote an easy birth to a strong, healthy baby [16]

### 1.3. Cordyla africana Lour. (Sunbird Tree, Wildemango, iGowane-Elikhulu, Umbhone)

*Cordyla africana* is a tall deciduous tree with wide-spreading crown, growing up to 25 m tall. The leaflets are thin and smooth, pale to dark green above, pale green below. Axillary flowers appear in September/October on short sprays, they are apetalous, but have showy, feathery golden stamens. The plant belongs to the legume family (Fabaceae), however the fruits are only pod-shaped when young, and become drupes when mature, golden yellow and glossy. The sap from the unripe fruit is used as gum [18]. There are no known medicinal uses of any parts of the tree, including the fruits. However, the wood has several uses including carvings, construction, furniture, tool handles, household utensils, flooring, joinery, interior trim, mine props, and toys [19]. Prospects for possible commercialization of this plant have not yet been explored.

### 1.4. Dovyalis caffra (Hook.f. & Harv.) Warb. (Kei-Apple, Kei-Appel, Motlhono, Umqokolo, Amaqokolo, Mukokolo)

The Kei-apple is a spiny evergreen shrub or tree growing up to 8 m tall. Leaves are fascicled or alternate on young shoots, leathery, and obovate or elliptic-rhomboid in shape. The plants are mostly dioecious with light green flowers produced in November to December. Male flowers arranged in fascicles and female flowers either in fascicles or solitary. The fruits are subglobose, minutely velvety and yellowish-orange in colour [20,21]. The edible fruit, for which *D. caffra* is commercialized, can be eaten fresh or dried and is used for making jelly, jam, liqueurs, sweets, and processed products [15]. In traditional medicine, fruit extract is used to promote gastrointestinal motility and inhibit tightening and shortening of the uterine muscles [22].

### 1.5. Dovyalis longispina Warb. (Coastal Kei-Apple, Natal Apricot)

*Dovyalis longispina* is a dioecious shrub or small tree, armed with long, thin spines. The branches bear alternate, leathery, obovate to rhomboid–elliptic leaves characterized by prominent veins on both surfaces. The leaves fall off when flowers appear, but re-grow immediately. Male flowers light green, female flowers yellow-green, both appearing from August to October and followed by orange or red fruits with white spots, the fruits are edible [23]. There are no known medicinal uses for any of the plant parts, but the plant is used for hedging.

### 1.6. Englerophytum magalismontanum (Sonder) T.D.Penn (Transvaal Milkplum, Stamvrug, Motlhatswa, Mohlatswa, Munombelo, Amanumbela, Umnumbela)

Transvaal milkplum is an evergreen, small to medium-sized tree that can reach heights up to 15 m, under forest conditions. The leaves are often crowded near the end of the branches, they are alternate, oblanceolate to obovate elliptic in shape, glossy green above and densely velvety, silvery, golden, or brown beneath, and have a prominent midrib. Flowering takes place from June-December, during which time small, pinkish-brown flowers form in dense clusters on the old leaf scars along branches. The fruits are ovoid with a sharp tip and bright red when ripe [24,25]. The edible fruit is used for making jam, syrup, and wine [13]. According to Rampedi [26], sensory analyses and market surveys suggested that *E. magalismontanum*, together with *Doyvalis caffra* and *Garcinia livingstonei* can be considered as good candidates for further development in the beverage industry.

### 1.7. Garcinia livingstonei T.Anderson (African Mangosteen, Afrika-Geelmelkhout, umPhimbi, uGobandlovu, Mmimbi, Mokongono, Mokononga)

African mangosteen is an evergreen shrub or tree, which can reach heights of 21 m, and it is characterized by short-twisted boles and long horizontal pendulous branches. The leaves occur in threes in a whorl, they are mostly oval or lanceolate in shape. The flowers are axillary and arranged in groups of 5–15 and greenish or yellow, they are followed by orange berries [27]. The edible fruits are used to make an alcoholic beverage [28]. The fruits are used for the treatment of mumps, while powdered root is used as an aphrodisiac [27] and to treat stomach problems in pregnant women [29]. Flavonoid compounds from *G. livingstonia* leaves were found to have moderate activity against *Enterococcus faecalis*, *Escherichia coli* [30], and *Mycobacterium smegmatis* [31].

### 1.8. Halleria lucida L. (Tree Fuchsia, White Olive, Notsung, Witolienhout, Witolyfhout, Umbinza, Indomela, Umbinza, Lebetsa, Murevhe)

*Halleria lucida* is an evergreen large shrub or multi-stemmed tree that can reach heights of up to 20 m in protected areas, and it is used mainly for ornamental purposes. The bark is longitudinally grooved and pale grey and brown in color. The plant is characterized by bright green leathery leaves and orange, red or yellow tubular flowers, which are produced from May to January [32]. The spherical fruits turn black when ripe. They have no known medicinal uses, however other parts of the plant are used medicinally, for example, the leaves are used for the treatment of skin conditions and earache [32].

### 1.9. Manilkara mochisia (Baker) Dubard (Lowveld milkberry (English) Mwambo (Shona)

*Manilkara mochisia* is an evergreen large shrub or a small to medium sized tree with a spreading crown, and it can grow up to 20 m tall. The obovate coriaceous leaves are formed in terminal rosettes at the ends of branches and short lateral shoots, and they are glossy green above and paler below. The greenish-yellow flowers form small clusters under the new leaves from October to December, followed by yellow, round fruits up to 18 mm long [33]. Although there are no recorded medicinal uses of the fruits, fruit pulp showed good antimicrobial activity against *Klebsiella pneumonia* [34]. The stem bark is used to treat mastitis.

### 1.10. Pappea capensis Eckl. & Zeyh. (Jacket Plum, Indaba Tree, Bushveld Cherry, Doppruim, Umqhokwane, Umvuna, Indaba, Ilitye, Umgqalutye, Mongatane, Mopsinyugane, Liletsa, Xikwakwaxu, Gulaswimbi)

*Pappea capensis* is an evergreen, small to medium monoecious (sometimes androdioecious) tree with a spreading crown, growing up to a height of 8 m. It comprises simple, oblong leaves, which are hard-textured and wavy. In young plants, the leaf margin is sharply toothed but becomes almost smooth at maturity. Yellowish-green flowers are produced in axillary catkins from September to March, followed by round green velvety fruits. At maturity, the fruits split open to reveal bright orange-red aril covering a shiny dark brown to black seed [35]. The fruit is eaten unripe or ripe and it is used for making fruit juice, preserves, jelly, vinegar, and an alcoholic drink. There are no known medicinal uses of the fruits, however the leaves and bark extracts are used for the treatment of baldness, ringworm, nosebleeds, chest complaints, eye infections, and venereal disease in Southern Africa [35]. An ethyl acetate fraction from the plant was found to have antimicrobial activity against *Bacillus subtilis*, *Escherichia coli*, *Staphylococcus aureus*, and *Candida albicans*, while isolated compounds from the plant exhibited antioxidant activity [36].

### 1.11. Parinari curaellifolia Planch ex. Benth. (Mobola Plum, Cork Tree, Hissing Tree, Grysappel, Bosappel, Mmola, Mobola, Muvhula)

The mobola plum is a medium-sized to large evergreen tree with an almost round canopy shaped like a mushroom, it can grow up to 26 m tall. The leaves are bicolored, and they are white-silver beneath and dark green-grey above; they are inwardly folded and have a characteristic prominent herringbone venation. The yellow, pink, or white flowers are produced in terminal panicles from July-November and followed by yellow ovoid fruits [37]. The edible fruit is used to make soft porridge, jam, and syrup [13]. In a previous study [37], the fruit pulp was found to contain relatively higher fat (4.51%) and vitamin C (66.06 mg/g) contents than the jam. The fruit is also used medicinally for the treatment of cancer, ear problems, sore eyes, fever, pneumonia, and microbial infections [13,38].

### 1.12. Phoenix reclinata Jacq. (Wild Date Palm, Wilde-Dadelboom, Mopalamo, Moséfa, Isundu)

*Phoenix reclinata* is a protected tree in South Africa. It is an evergreen palm that grows to average heights of 3–6 m tall, although it can grow up to a maximum of 12 m. It has slender, leaning stems, with spiny leaves forming a crown at the top of the stems. The plants are dioecious, and the flowers are produced from August-October, followed by orange-brown, oval fruits borne on drooping branches in February-April [39,40]. The edible fruits are eaten cooked or raw, and used for making processed products [15]. They have no known medicinal uses, however unspecified parts of the plant are used to treat pleurisy and the spines are used for unspecified medicinal purposes [39]. The sap from the plant is used for making palm wine. The leaves have several ethnobotanical uses including making bags, baskets, hats, and mats [40].

### 1.13. Syzygium cordatum Hochst. (Water Berry, Waterbessie, Waterbessieboom, Waterboom, Waterhout, Umdoni, Umswi, Umjomi, Mawthoo, Motlho, Mutu, Muhlwa)

The water berry is an evergreen tree with a round spreading crown, up to 20 m tall. The bluish-green, gland-dotted leaves are leathery, round, smooth, and reddish when young. The creamy-white, pale pink, or yellow flowers produced on terminal heads are characterised by numerous fluffy stamens. The fruit is a round fleshy berry and turns red to dark purple or black when ripe [41]. The edible fruit is used for making an alcoholic beverage [13], jam, jelly, and processed products [15]. There are no known medicinal uses for the fruits, but the stem bark, leaves, and roots have numerous medicinal uses [42]. Correspondingly, extracts from the different plant parts have been shown to have several pharmacological properties including antibacterial, antidiabetic, antifungal, anti-inflammatory, and antiplasmodial activities [42].

### 1.14. Syzygium guineense DC. (Woodland Waterberry, Waterpear, Waterpeer, Umdoni)

*Syzygium guineense* is medium-sized to large evergreen tree with a round crown and can grow up to heights of 30 m. The leaves are purplish-red when young, turning yellowish-green or glossy as they mature. Creamy white flowers are produced from August-December, followed by dark purple fruits from December to April [43]. The edible fruit has a very short shelf life and should therefore be consumed soon after harvesting, it is used to make a beverage or vinegar. Almost all plant parts have several uses in traditional medicine and the fruit specifically, is used for the treatment of dysentery, the bark is however reported to be toxic [44]. Extracts from different parts have exhibited antimicrobial activity against *Bacillus subtilis*, *Escherichia coli*, and *Shigella sonnei* [44].

## 2. Results and Discussion

### 2.1. Literature Survey

The botanical information of the identified wild indigenous Southern African plants is presented in Table 1, as presented in Sibiya et al. [45]. Table 2 shows the essential amino acids present in these plants based on previous studies. The results show that Anacardiaceae is one of the most utilized families (7 spp.), however it showed a large nutrient knowledge gap, for example, none of the *Searsia* species (i.e., *Searsia undulata*, *Searsia discolor*, *Searsia pentheri,* and *Searsia dentata*) have previously been tested for their nutritional content. Nevertheless, the very well-known fruit, *Sclerocarya birrea* (marula), was tested the most in this family. This review shows that this fruit has been of interest for many years, as exemplified by the number of publications covering its nutrition [46,47,48,49,50,51,52]. Cucurbitaceae, which has the same number of species as Anacardiaceae, had the highest number of species (4) previously studied. Surprisingly, fruits belonging to 12 of the 35 families recorded in this study had not previously been tested for any nutritional content. Although the family Fabaceae is the second most economically important family in the world, it is represented only by *Cordyla africana*. In this study, the word ‘fruit’ is used in the common usage sense rather than the scientific sense, therefore leguminous plants were excluded. Furthermore, the nutrients, specifically proteins, tend to be concentrated in the leaves and seeds [53].

### 2.2. Proximate Composition

Table 3 displays the proximate values showed at a significant difference *p* ≤ 0.05, obtained from the 15 fruit samples. Statistically, the moisture content showed a significant difference, ranging from 19.61 g/100 g to 88.11 g/100 g. The moisture content of *Carpobrutus edulis* was significantly lower than that of other fruits. These results may be attributed to the high content of pectin, which is a very sticky gel-like substance that did not evaporate at the prescribed temperature [97]. *Englerophytum magalismontanum* showed the highest moisture content (88.11 g/100 g), followed by *Syzygium guineense* (82.41 g/100 g) and *Syzygium cordatum* at 81.78 g/100 g. The levels of moisture obtained from the pulp and skin of *S. guineense* and *S. cordatum* were highly comparable (expressed by consecutive letter superscripts ‘m’ and ‘n’), possibly because the fruits are from the same genus, and the fact that their common name ‘water-berry’ advocates their high water content. The results obtained show that the stamvrug and the water-berries could make good fruit juice products.

Statistically, *Carissa macrocarpa* was observed to have the highest ash content at 20.28 g/100 g and *Cordyla africana* to have the lowest at 1.97 g/100 g. For other species, this value significantly varied between 2.05 g/100 g to 8.76 g/100 g. The results for *S. guineense* and *P. capensis* were statistically similar (expressed by a ‘c’ superscript), with a concentration of 3.34 g/100 g and 3.42 g/100 g, respectively. A possible explanation for the similar amounts of ash in the two species could be that they have equivalent mineral compositions, since ash is the remaining inorganic part of a plant after combustion. However, a study by Sibiya et al. [49] showed that there are significant differences in mineral composition, for example, *P. capensis* had much higher amounts of aluminum, calcium, potassium, and selenium than *S. guineense*, while the latter had higher contents of magnesium and slightly higher quantities of lead. The levels of fat for the 15 fruit samples ranged significantly between 0.003 and 7.74 g/100 g. *Syzygium guineense* showed the highest fat content than all other fruits at 7.74 g/100 g, while *Dovyalis caffra* from Nelspruit recorded the lowest fat content of 0.003 g/100 g. Overall, the results obtained are in agreement with [98], proving that most fresh fruits are a poor source of fat.

The proximate fiber value was the third-highest amount captured in this study, at 29.89 g/100 g, and it was recorded in *Phoenix reclinata*. The findings, shown in Table 1 indicate that wild edible indigenous fruits could be a good component for weight loss and reducing the risk of cardiovascular disease. The Recommended Daily Allowance (RDA) for fiber is 18 to 35 g [99], implying that 100 g of *P. reclinata* fruit is adequate to meet the daily fiber requirements of the body. As shown in Table 3, the protein levels of the fruits ranged significantly from 0.004 to 6.98 mg/100 g.

The highest protein value was recorded in *Halleria lucida* (6.98 g/100 g), followed by *Pappea capensis* (4.33 g/100 g) and *P. reclinata* (4.08 g/100 g). According to AOAC [99], the Recommended Dietary Allowance (RDA) of protein for children, adult males, adult females, pregnant women, and lactating mothers are 28, 63, 50, 60, and 65 g, respectively. If 100 g of *H. lucida* provides 6.98 g of proteins, this indicates that fruits could be poor sources of proteins. The quality of a protein is determined by evaluating its composition of the essential amino acids, digestibility, and bioavailability of the amino acids [100]. According to the joint Food Agricultural Organisation/WHO Expert Consultation on Protein Quality Evaluation, the protein digestibility corrected amino acid score (PDCAAS) method should be used to evaluate protein quality [101], whereby only the limiting amino acids are considered. In this calculation, a protein source with a score of 1 is regarded as having the highest quality and those with scores below 1 do not have enough essential amino acids. In the current study, only the amino acid composition of the 15 samples was assessed.

The energy values were measured from 18.51 to 169.10 kJ/100 g. *Manilkara mochisia* was well above all fruits, but not far from *P. capensis* at 163.31 kJ/100 g, illustrating that these fruits have high calorific value. According to Johnson et al. [102], high energy density foods tend to include foods that are high in fat and have low water content. This statement was found correct, as *P. capensis* showed a high content for fat (5.11 g/100 g), and a low moisture content of 45.63 g/100 g.

### 2.3. Amino Acid Composition

Of the 60 species recorded, only 5 had previously been evaluated for their amino acid composition. The results of amino acid quantities of the studied wild indigenous are shown in Table 4. In the current study, arginine and serine were the most abundant amino acids in *D. longispina* and the highest recorded compared to all fruits, at 3.31 and 1.14 g/100 g, respectively. Although classified as a non-essential amino acid (NEAA), arginine plays an important role in the removal of ammonia from the body, during cell division and in synthesizing nitric oxide for blood pressure regulation [103,104,105]. Research has shown that “young and gestating mammals cannot synthesize sufficient amounts of all NEAA to support maximum embryonic/fetal survival, neonatal growth, as well as vascular and intestinal health” [106] (p.31). The high *H. lucida* protein concentration was exhibited by a high histidine concentration, at 1.56 g/100 g, thus showing alignment between the proximate and the results of the amino acid. This suggests that 100 g of *H. lucida* exceeds the required amounts of 14 mg/kg (0.0014 g/100 g) for histidine. Histidine has important cellular functions as it is a precursor for several hormones and other critical metabolites, it is also chelator of metal ions such as copper, zinc, manganese, and cobalt [107].

*Halleria lucida* also had the highest amounts of isoleucine (0.30 g/100 mg), leucine (0.47 g/100 g), phenyalanine (0.31 g/100 g), and valine (0.39 g/100 g). In all instances, the amounts exceed the WHO RDAs. lsoleucine, leucine, and valine are essential amino acids known as branched chain amino acids (BCAAs), they have the same enzymatic steps in their pathway [108]. They are involved in several physiological and metabolic functions, for example, they participate in glucose transportation and metabolism, mammary health, and intestinal barrier function and absorption [108]. However, leucine has more prominence because of its function in the activation of the mammalian target of rapamycin signaling pathway [108,109]. In addition, leucine enhances energy homeostasis, provides skeletal muscles with an increased flux of lipids, and supplies energy substrates to support protein synthesis [110]. Deficiencies of BCCAs can lead to a number of disorders such as maple syrup urine disease, osteoporosis, Barth syndrome, Costeff optic atrophy, and propionic acidemia [108]. Isoleucine plays a vital role in physiological processes including fatty acid metabolism, transportation of glucose, growth, and immunity [111]. Phenylalanine is used by the body for the synthesis of catecholamines and melanin and also serves as a precursor of the amino acid tyrosine [112].

The requirement for lysine has received the most attention given its importance as the likely limiting amino acid in staple foods, such as wheat. The highest lysine captured was 0.77 g/100 g for *P. capensis*. The jacket-plum meets and exceeds the required daily intake (0.0003 g/100 g or 3 mg/kg) recommended by the WHO. The lysine content of all fruits tested is significantly higher than the lysine content required within 24 h in adults. The biological functions of lysine include support for healthy growth and development as well as the maintenance of healthy immune function, particularly concerning antiviral activity [113]. *Pappea capensis* also had the highest quantities of methionine (0.15 g/100 mg) and threonine (0.31 g/mg), however the value for methionine is much lower than the FDA recommended daily allowance but is equivalent to the WHO RDA. The amount of threonine in *P. capensis* is higher than the daily allowances recommended by both the FDA and WHO. Methionine is a sulfur-containing amino acid and in addition to protein synthesis, it serves as a donor of methyl and aminopropyl in transmethylation processes and in the synthesis of polyamides, respectively [114]. Thrionine promotes normal growth by helping in maintaining proper protein balance in the body, and it also supports cardiovascular, liver, central nervous, and the immune system. Furthermore, threonine is involved in the synthesis of other amino acids, i.e., glycine and serine [115]. *Englerophytum magalismontanum* was found to contain the highest tyrosine content (0.38 g/mg), which is higher than the recommended daily intake by the FDA and WHO. Tyrosine, together with phenylalanine and tryptophan, are aromatic amino acids and they serve as precursors for the monoamine neurotransmitters, serotonin and cathecholamines in the brain [116].

**Figure 2:**
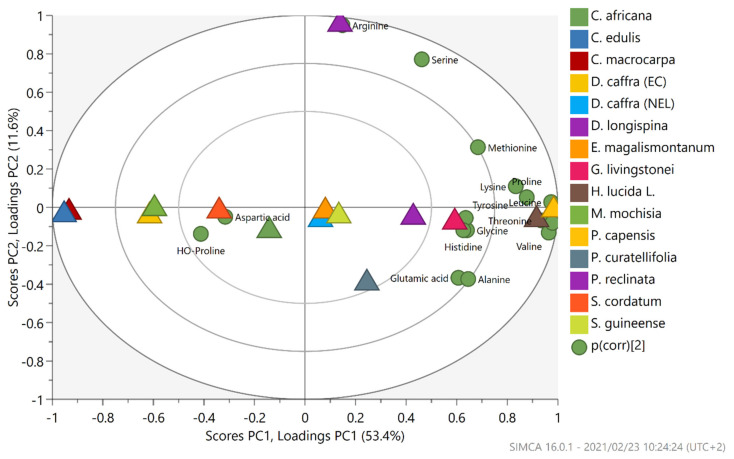
Principal Component Analysis (PCA) multivariate projection score and loading scatter plots of amino acids composition in wild indigenous fruits of Southern Africa. Fruit samples color coded as: *Cordyla africana* (dark green), *Carpobrotus edulis* (blue), *Carissa macrocarpa* (deep red), *Dovyalis caffra*-EC (mustard), *Dovyalis caffra*-NEL (light blue), *Dovyalis longispina* (purple), *Englerophytum magalismontanum* (light orange), *Garcinia livingstonei* (Red), *Halleria lucida* (brown), *Manilkara mochisia* (green), *Pappea capensis* (yellow), *Parinari curatellifolia* (dark blue), *Phoenix reclinata* (Orchid purple), *Syzygium cordatum* (orange), and *Syzygium guineense* (light green).

Using the data obtained for amino acid composition of selected wild African edible fruits, a Principal Component analysis (PCA) model was employed to summarize the variation in amino acids present in the different wild fruit samples analyzed. Figure 2 shows the projection of scores of wild fruit samples (with different color codes) and illustrates loading projections of amino acids identified. The two principal components described 65% of the total variation of amino acids in wild fruit samples and showed separation based among fruit samples studied. The first principal component (PC1) explained 53.4% of the total variation where samples on the left side of the plot are differentiated from those on the right side. It was observed that most amino acids were concentrated on the right side of the plot, which indicates that wild fruit samples on the far right of the plot had higher levels of these amino acids than those on the far left side of the plot (Figure 1). This means that most amino acids, namely, lysine, methionine, proline, tyrosine, valine, glycine, alanine, glutamic acid, leucine, histidine, and threonine were predominant in *Halleria lucida*, *Garcinia livingstonei*, *Phoenix reclinata* and *Pappea capensis*. Notably, the majority of these are considered essential amino acids because they cannot be made by the human body and can only be obtained from diets. Therefore, when these wild fruit are consumed or used together with other foods, they can contribute to the nutritional quality. The second principal component (PC2) added 11.6% to the explanation of variation among samples and mostly separated *Dovyalis longispina* (upper side of the plot) with higher levels arginine and serine, while *Parinari curatellifolia* (bottom side of the plot) was associated with high concentration of glutamic acid and alanine.

## 3. Materials and Methods

### 3.1. Literature Survey

The literature survey was compiled from information collected from various search engines and databases including EBSCO, Google Scholar, JSTOR, and Scopus, which were searched using the scientific and common names of the species as well as keywords such as chemical composition, nutritional value, and proximate analysis. The data were then summarized into a table comprising scientific name, common name, nutrient composition, and references. Species with information gaps were reviewed three times to confirm accuracy.

### 3.2. Fruit Samples for Evaluation

#### 3.2.1. Selected Fruits for Analysis

Fourteen wild edible fruits were selected (based on availability and knowledge gap) and collected randomly across Southern Africa. The species tested were *Carissa macrocarpa*, *Carpobrotus edulis*, *Cordyla africana*, *Dovyalis caffra* (two samples from different localities), *D. longispina*, *Englerophytum magalismontanum*, *Garcinia livingstonei*, *Halleria lucida* L., *Manilkara mochisia*, *Pappea capensis*, *Parinari curatellifolia*, *Phoenix reclinata*, *Syzygium cordatum,* and *S. guineense*.

#### 3.2.2. Processing of Fruits

The edible parts of the fruits (pulp and peel) were carefully separated from the seed. The material was homogenized using a mortar and pestle [9]. The fruits were then freeze-dried, except for the samples that were analyzed for moisture content.

### 3.3. Proximate Analysis

#### 3.3.1. Moisture Content Determination

Moisture was determined using the procedure described in the American Association of Cereal Chemists (AACC) Method 44–15A [117]. Moisture crucibles were dried in a forced draught oven at 103 °C for 1 h. The crucibles were then cooled in a desiccator for about 10 min. The crucibles were weighed, and 2 g of sample was weighed into the crucibles and dried in a forced draught oven for 4 h at 103 °C. The samples were cooled for 10 min and weighed. The moisture content percentage was calculated as follows:

$$\%\mathrm{moisture}=\;\frac{[\left(\mathrm{mass}\;\mathrm{food}+\mathrm{tin}\right)\;-\;\left(\mathrm{mass}\;\mathrm{tin}\right)\;-\;\left[\left(\mathrm{mass}\;\mathrm{dry}\;\mathrm{food}+\mathrm{tin}\right)\;-\;\left(\mathrm{mass}\;\mathrm{tin}\right)\right]x\;100}{\left(\mathrm{mass}\;\mathrm{food}+\mathrm{tin}\right)-\mathrm{mass}\;\mathrm{tin}}$$

#### 3.3.2. Protein Content Determination

The micro Kjeldahl method described by the Association of Official Analytical Chemists [7] was used. Two grams (2 g) of each sample was mixed with 10 mL of concentrated sulfuric acid in a heating tube. One tablet of selenium catalyst was added to the tube and the mixture was heated inside a fume cupboard. The digest was transferred into a 100 mL volumetric flask and made up with distilled water. Ten-milliliter portion of the digest was mixed with an equal volume of 45% NaOH solution and poured into a Kjeldahl distillation apparatus. The mixture was distilled, and the distillate was collected into a 4% boric acid solution, containing 3 drops of indicator. A total of 50 mL distillate was collected and titrated as well. The sample was duplicated, and the average value was taken. The nitrogen content was calculated and converted to percentage protein by using a protein conversion factor of 6.25. This was given as:

$$\%nitrogen=\frac{\left(100\;\times \;W\;\times \;N\;\times \;14\;\times \;\mathrm{Vf}\right)T}{100\;\times \;\mathrm{Va}}$$

where W = Weight of the sample, N = Normality of the titrate (0.1 N), Vf = Total volume of the digest = 100 mL, T = Titre value and Va = Aliquot volume distilled.

#### 3.3.3. Ash Content Determination

To determine the ash content, which is the substance remaining after oxidative combustion of all the organic matter in food, the AACC method 08–01 [118] was used. Crucibles were dried in an oven at 100 °C for 5 h and allowed to cool to be weighed. Two grams of each sample was weighed into the crucibles. The crucibles with samples were then placed on a tripod and heated until the samples are charred. The charred samples were then put in a muffle furnace and heated at 550 °C for 5 h. The samples were removed and cooled in a desiccator until room temperature and then weighed. Percentage ash was calculated as follows:

$$\%\;\mathrm{ash}=\;\frac{\left(\mathrm{mass}\;\mathrm{ash}+\mathrm{crucible}\right)-\left(\mathrm{mass}\;\mathrm{crucible}\right)\times \;100}{\left(\mathrm{mass}\;\mathrm{food}+\mathrm{crucible}\right)-\left(\mathrm{mass}\;\mathrm{crucible}\right)}$$

#### 3.3.4. Fat Content Determination

Crude fat was determined by extracting the sample using petroleum ether, according to the AACC method 30–25 [119] of the Soxhlet test apparatus. The petroleum ether was then removed from the collection flask at low-temperature volatilization before oven drying. The residue fat was dried in an oven at 100 °C for 30 min. Percentage of fat was calculated using the following formula:

$$\%\;\mathrm{fat}=\;\frac{\left(\mathrm{mass}\;\mathrm{of}\;\mathrm{beaker}+\mathrm{mass}\;\mathrm{of}\;\mathrm{extracted}\;\mathrm{fat}\right)-\left(\mathrm{mass}\;\mathrm{of}\;\mathrm{beaker}\right)\times \;100}{\mathrm{mass}\;\mathrm{of}\;\mathrm{sample}}$$

#### 3.3.5. Fiber Content Determination

Crude fiber was determined by first weighing the filter bag (W_1_), then 0.5 g of dried sample (W_2_), that was ground to pass a 1 mm screen, and directly added into a filter bag. A blank bag was weighed and included in digestion, to determine blank bag correction (C_1_). The bags were sealed within 0.5 cm from the pen edge, using the heat sealer. The bags were placed in a bag suspender. About 2000 mL of temperature acid detergent solution was added into the ANKOM Fiber Analyzer vessel and the suspender was covered. The fiber analyzer vessel was set for 60 min. Approximately 2000 mL of hot water was added, and the samples were agitated again. The samples were rinsed for 3–5 min with water until a neutral pH was reached. Excess water was pressed out from the bags. The bags were then placed in a beaker and covered with acetone for 3 min. The bags were removed and pressed again to remove excess acetone. The bags were dried in the oven at 105 °C, for at least 2 h. The bags were cooled to ambient temperature and weighed (W_3_).

$$\%\;\mathrm{ADF}\;\left(\mathrm{as}-\mathrm{is}\;\mathrm{basis}\right):=\frac{\left[W_{3}-\left(W_{1}\;\times {\;C}_{1}\right)\right]\times \;100}{W_{2}}$$

#### 3.3.6. Carbohydrates Determination

The available carbohydrate content in the samples was calculated by difference as follows: % carbohydrate = 100 − (% moisture +% ash +% crude protein +% crude fat +% crude fiber).

### 3.4. Amino Acid Analysis

Amino acid compositions of the fruit samples were determined at the Agricultural Research Council (ARC)—Irene Analytical Research Laboratory, Pretoria, South Africa, using high-performance liquid chromatography (HPLC), following the procedure of [120]. Briefly, milled samples (700 mg) were hydrolyzed using an equal volume of 6 N HCL solution and an internal standard (β-guanidinopropionic acid), for 24 h at 115 °C and left to cool. The hydrolysate was transferred to an Eppendorf tube and centrifuged at 3000 rpm for 10 min, after which the supernatant (protein hydrolysate) was filtered (0.45 mm). The protein hydrolysate was dried under nitrogen gas and derivatized using FMOC reagent (9-flourenylmethyl chloroformate) and borate buffer. After a few seconds, the mixture was extracted with pentane. The derivatized extract was analysed on HPLC using fluorescence detectors (Schoeffel FS 970, Perkin-Elmer LS-4, and Shimadzu RF-530 at excitation and emission wavelengths of 260 and 313 nm respectively). A mixture of HPLC grade acetonitrile, methanol, and acetic acid (10:40:50, *v*/*v*/*v*) was used as the eluent and varied linearly to acetonitrile: acetic acid (50:50, *v*/*v*) over 90 min. Oven temperature was kept at 40 °C while the gradient flow initiated at 3 min at 1.3 mL/ min flow rate. The flow rate was later increased to 2 mL/min for 0.5 min at the end of the gradient. Using calibration curves from amino acid standards (Sigma-Aldrich, Darmstadt Germany), the concentration of the amino acids in the samples was then determined.

### 3.5. Determination of Energy Value

The sample calorific value was calculated in kilocalories (kcal) by multiplying the energy factor composition (4, 4, and 9) by percentage protein, carbohydrates, and fats respectively [121,122]. The conversion factors are equivalent to the physiological energy, which is the energy value remaining after losses due to digestion and metabolism [123].

### 3.6. Statistical Analysis

Duplicate samples were prepared, and analyses were done in triplicate. The results were statistically analyzed by using the Statistica statistical software for proximate analysis. Results were shown as means ± standard deviation. One way Analysis of Variance (ANOVA) with Fisher Least Significant Difference (LSD) was used to analyze the means between the 15 fruit samples type. The difference of mean values was analyzed by Fisher Least Significant Difference (LSD) tests. The significance difference of the samples was determined at a confidence level above 95% (*p* < 0.05). Principal component analysis (PCA) was performed using the SIMCA statistical software version 14.1 (Umetrics, Umea, Sweden) to analyze the amino acid composition data.

## 4. Conclusions

Based on the literature survey, it can be depicted that most of the species that have been successfully commercialized were supported with adequate background research, highlighting some of their marketable properties. The best macronutrient combination of fruits to meet daily requirements would be *Phoenix reclinata* (fibre), *Syzygium guineense* (fat), and *Halleria lucida* (protein). All the species studied were found to have lower amounts of indispensable amino acids, except for *H. lucida,* which contains higher amounts of histidine than the recommended dietary allowance. *Manilkara mochisia* yielded the highest amount of energy, however a daily consumption of 5 kg by an average adult would be required to meet the RDA value of 2000 kcal. In addition, all the tested fruits would need to be taken in large quantities to meet the recommended daily allowance for fat. One hundred grams of *Englerophytum magalismontanum* contain 0.08 liters of moisture, which is more or less equivalent to an average apple, however the fruits cannot be used as the sole source of moisture to meet the recommended daily intake of 2.1–2.6 liters of water for adults. Although the fruits of *Phoenix reclinata* are smaller than the commercial date palm, the plant has potential as it is already well-known. *Halleria lucida* also had the highest carbohydrate content among all the fruits evaluated and it is also easy to grow, however it is not a very well-known plant and therefore may not be easy to market. The commercial potential of any of the plants evaluated in this study would require substantial amounts of research. Future research should include evaluation of protein quality as determined by the bioavailability and digestibility of the amino acids. Other factors that should be considered include palatability, size, yield, sensory analysis, and shelf life.

## Figures and Tables

**Table 1:** List of wild indigenous Southern African plant species with edible fruits, their common names, known nutritional value, as well as references.

Species	Common Name	Proximate (g/100 g)						Vitamin C (mg/100 g)	Reference
		P	F	Fat	A	C	M		
Anacardiaceae									
*Harpephyllum caffrum* Bernh.	Wild plum	0.7	1.7	0.2	0.8	9.1	87.5	-	[54,55,56]
*Lannea edulis* (Sond.) Engl.	Wild grape	6.5	5.5	9.7	2.4	75.9	-	14	[57]
*Sclerocarya birrea* (A.Rich.) Hochst.	Marula	30.1 ± 0.2	10.5 ± 2.7	58.3 ± 1.3	3.8 ± 0.0	3.7	25.3%	400	[46,47,48,49,50,51,52,56,57,58,59]
*Searsia undulata* (Jacq.) T.S.Yi, A.J.Mill. & J.Wen.	Taaibos	-	-	-	-	-	-	-	-
*Searsia discolor* E. Mey. ex Sond.	-	-	-	-	-	-	-	-	-
*Searsia pentheri* Zahlbr.	-	-	-	-	-	-	-	-	-
*Searsia dentata* Thunb.	Nana berry	-	-	-	-	-	-	-	-
Annonaceae									
*Annona senegalensis* Pers.	Wild custard apple	8.80%	17.6%	24%	12.10%	25.3%	7.79%	-	[55,60]
*Hexalobus monopetalus* (A.Rich.) Engl. & Diels	Shakama plum	-	-	-	-	-	-	-	-
Apocynaceae									
*Ancylobotrys capensis* (Oliv.) Pichon	Wild apricot	1.0	0.8	0.2	0.6	33.1	64.0	-	[56,58]
*Carissa macrocarpa* (Eckl.) A.DC.	Natal plum	0.74 ± 0.03	-	3.53 ± 0.01	0.50 ± 0.09	100.3 ± 0.2	78.45%	168.36	[61,62,63]
*Sarcostemma viminale* (L.) R.Br.	Caustic bush	-	-	-	-	-	-	-	-
Arecaceae									
*Phoenix reclinata* Jacq	Wild date palm	0.2	-	-	0.4	-	22 + 109	6.5	[64]
Asteraceae									
*Chrysanthemoides monilifera* (L.) Norl.	Bietou	-	-	-	-	-	-	-	-
Capparaceae									
*Boscia albitrunca* (Burch.) Gilg & Gilg-Ben.	Emigrant’s tea/Caper bush	-	-	-	-	-	-	-	-
Caryophyllaceae									
*Pollichia campestris* Aiton	Waxberry	-	-	-	-	-	-	-	-
Celastraceae									
*Salacia kraussii* (Harv.) Harv.	Ibontsi	3.7 ± 0.00	-	1.5 ± 0.6	4.8 ± 0.2	-	-	-	[54,57]
Chrysobalanceae									
*Parinari curatellifolia* Benth.	Mobola plum	3.0%	5.5%	1.5%	1.8%	88.2%	-	75	[9,65,66]
Clusiaceae									
*Garcinia livingstonei* T.Anderson	African mangosteen	0.65 ± 0.02%	21.13 ± 0.02%	2.56 ± 0.06%	1.76 ± 0.04%	95.02 ± 0.01%	0.45 ± 0.13%	7.2	[67]
Cucurbitaceae									
*Acanthosicyos horridus* Welw. ex Hook.f.	Nara	31%	-	-	-	-	-	-	[68]
*Acanthosicyos naudiniana* (Sond.) C.Jeffrey	Gemsbok cucumber	-	-	-	-	-	-	-	-
*Citrullus lanatus* (Thunb.) Matsum. & Nakai	Tsamma	0.44 ± 0.05	0.19 ± 0.01	0.15 ± 0.01	0.21 ± 0.01	7.19 ± 0.05	91.92 ± 0.01	-	[69,70,71]
*Coccinia sessilifolia* (Sond.) Cogn	Red gherkin	2.1	1.3	0.2	0.2	13	82.3	25	[72]
*Cucumis anguria* L.	Gherkin	-	-	-	-	-	-	-	-
									
*Cucumis metuliferus* E.Mey. ex Naudin	Jelly melon	1.8	1.1	1.3	-	8	88.97	5.3	[73]
*Cucumis myriocarpus* Naud	-	-	-	-	-	-	-	-	
Ebenaceae									
*Diospyros mespiliformis* Hochst. ex A.DC.	Jackal berry	10.26 ± 0.01%	2.00 ± 0.13%	3.51 ± 0.18%	3.00 ± 0.20%	75.23 ± 0.40%	6.01 ± 0.00%	-	[74,75]
*Euclea crispa* (Thunb.) Gϋrke	Bush guarri	-	-	-	-	-	-	-	-
Euphorbiaceae									
*Bridelia mollis* Hutch.	Velvet sweet berry	-	-	-	-	-	-	-	-
*Uapaca kirkiana* Müll.Arg.	Sugarplum	1.8%	8.4%	1.1%	2.2%	86.5%	-	-	[9,59]
Fabaceae									
*Cordyla africana* Lour.	Wild mango	-	-	-	-	-	-	75.6	[58]
Flacourtiaceae									
*Dovyalis caffra* (Hook.f. & Harv.) Sim	Kei-apple	0.4	0.7	0.4	0.5	4.7	85.9	275.88 mg/g	[58,76]
*Dovyalis longispina* (Harv.) Warb.	Natal Kei-apple	-	-	-	-	-	-	-	-
*Flacourtia indica* (Burm.f.) Merr.	Governor’s plum	4.2%	5.7%	3.6%	5.7%	80.7%	-	-	[9]
Hydnoraceae									
*Hydrona africana* Thunb.	Jakkalskos	-	-	-	-	-	-	-	-
Iridaceae									
*Romulea rosea* (L.) Eckl.	Frutangs	-	-	-	-	-	-	-	-
Lauraceae									
*Cryptocarya wyliei* Stapf	Red quince	-	-	-	-	-	-	-	-
Loganiaceae									
*Strychnos spinosa* Lam.	Green monkey apple	5.4%	17.6%	31.2%	42.1%	28.7%	78.8%	-	[9,56,58,60,76]
Malvaceae									
*Azanza garckeana* (F. Hoffm) Exell & Hillc.	Snot apple	12.0%	45.3%	1.1%	35.2%	28.4%	13.54%	30.8 mg/100 g	[5,9,77,78,79,80,81]
*Morella serrata* Lam.	Lance-leaf wax berry	-	-	-	-	-	-	-	-
Mesembryanthemaceae									
*Carpobrotus edulis* (L.) L.Bolus	Sour fig	-	-	-	-	-	-	-	-
Moraceae									
*Ficus sycamorus* L.	Sycamore fig	5.6 ± 0.2%	0.9%	8.9 ± 0.5%	4.4 ± 0.4%	25.3 ± 1.1%	55.8 ± 0.3%	-	[82]
Myrtaceae									
*Syzygium cordatum* Hochst.ex C.Krauss.	Water-berry	5.91%	1.7%	7.7%	2.3%	63.9%	23.8%	-	[42,56,83]
*Syzygium guineense* (Willd.) DC.	Water-berry	10.1%	30.3%	4.0%	7.1%	-	-	-	[84]
Olacaceae									
*Ximenia americana* L.	Blue sour plum	7.26 ± 0.2%	3.0 ± 0.01%	13.0 ± 0.02%	10.5 ± 0.1%	-	64 ± 0.2%	21.12 ± 0.01%	[57,85,86]
*X. caffra* Sond.	Sour plum	3.1	0.7	1.3	1.4	26.3		49.2	[9,87]
Polygalaceae									
*Nylandtia spinosa* (L.) Dumort.	Skilpadbessie	-	-	-	-	-	-	-	-
Rhamnaceae									
*Berchemia discolor* (Klotzsch) Hemsl.	Brown ivory	16.8%	9.57%	0.33%	4.25%	19.09%	59.53%	45.45%	[60,88]
*Ziziphus mucronata* Willd.	Buffalo-thorn	35.10 ± 0.58%	11.04 ± 0.88%	27.40 ± 0.11%	2.85 ± 0.23	23.61 ± 0.63%	4.10 ± 0.32%	-	[46,89]
Rosaceae									
*Rubus rigidus*	Wild bramble	-	-	-	-	-	-	-	-
Rubiaceae									
*Vangueria infausta* Burch.	Wild medlar	4.7 ± 0.4	26.3 ± 0.9	0.7 ± 0.2	5.7 ± 0.4	78.10%	80.70%	67.70%	[9,53,85,90,91]
Sapindaceae									
*Pappea capensis* Eckl. & Zeyh.	Jacket-plum	-	-	-	-	-	-	-	[35,36,92]
Sapotaceae									
*Englerophytum magalismontanum* (Sond.) T.D.Penn.	Stamvrug	-	-	-	-	43	-	20	[59,93]
*Manilkara mochisia* (Baker) Dubard	Lowveld milkberry	-	-	-	-	-	-	-	-
*Mimusops zeyheri* Sond.	Transvaal red milkwood	9.3%	-	-	4.1%	2.0%	-	-	[94]
Scrophulariaceae									
*Halleria lucida* L.	White olive	-	-	-	-	-	-	-	-
Solanaceae									
*Solanum retroflexum* Dunal	Nightshade	-	-	-	-	-	-	-	-
Tiliaceae									
*Grewia flava* DC.	Velvet raisin bush	-	-	-	-	82.1%	-	-	[62]
Verbenaceae									
*Lantana rugosa* Thunb.	Chameleon’s berry	-	-	-	-	-	-	-	-
Vitaceae									
*Rhoicissus tomentosa* (Lam.) Wild & R.B. Drumm.	Wild grape	1.4	-	-	-	-	-	-	[95]
*Rhoicissus tridentata* (L.f.) Wild & Drum.	Bushman’s grape	-	-	-	-	-	-	-	-

- = not tested; A = ash, C = carbohydrates, F = fibre, M = moisture, P = protein.

**Table 2:** Quantities of essential amino acids recorded in wild indigenous Southern African fruits and references.

Taxon	Amino Acid (mg/100 g)									
	Histidine	Isoleucine	Leucine	Lysine	Methionine	Phenylanine	Threonine	Tyrosine	Valine	Ref.
*Annona senegalensis*	-	25	20	30	15	25	15	39	25	[57]
*Azanza garckeana*	3.67	4.98	12.01	11.78	2.00	8.00	4.78	4.89	6.00	[81]
*Diospyros mespiliformis*	2.3	3.7	5	-	1	3.3	3	2	4.3	[75]
*Mimusops zeyheri* (seed)	0.37 ± 0.06	0.38 ± 0.01	0.58 ± 0.01	0.64 ± 0.04	0.12 ± 0.01	0.34 ± 0.01	0.44 ± 0.00	0.71 ± 0.13	0.49 ± 0.00	[94]
*Sclerocarya birrea* (nut)	6.83	12.93	16.72	7.73	7.45	11.91	6.03	6.34	13.07	[96]

**Table 3:** List of wild indigenous fruit plants and proximate value, as well as Recommended Daily Allowance (RDA) range values in g/day).

Species	Proximate g/100 g						Energy kJ/100 g
	Ash	Fat	Fibre	Moisture	Protein	Carbohydrates	
*Carissa macrocarpa*	20.28 ± 0.12 ^l^	0.87 ± 0.05 ^h^	4.68 ± 0.22 ^f^	52.48 ± 0.00 ^e^	1.96 ± 0.00 ^f^	21.57 ± 0.01 ^l^	101.95 ± 0.00 ^k^
*Carpobrotus edulis*	8.76 ± 0.00 ^k^	0.21 ± 0.00 ^b^	11.46 ± 0.00 ^l^	19.61 ± 0.03 ^a^	3.45 ± 0.00 ^j^	10.97 ± 0.00 ^e^	59.57 ± 0.00 ^e^
*Cordyla africana*	1.97 ± 0.00 ^a^	3.55 ± 0.00 ^l^	3.06 ± 0.00 ^c^	71.48 ± 0.01 ^i^	0.004 ± 0.02 ^a^	19.94 ± 0.00 ^j^	111.73 ± 0.00 ^l^
*Dovyalis caffra* (EC)	3.90 ± 0.00 ^f^	0.73 ± 0.00 ^g^	3.44 ± 0.00 ^d^	79.30 ± 0.00 ^k^	0.83 ± 0.00 ^b^	11.80 ± 0.00 ^h^	57.09 ± 0.00 ^d^
*Dovyalis caffra* (NEL)	2.50 ± 0.00 ^b^	0.003 ± 0.02 ^a^	2.48 ± 0.00 ^b^	79.26 ± 0.00 ^j^	1.56 ± 0.00 ^c^	14.20 ± 0.02 ^i^	64.27 ± 0.00 ^f^
*Dovyalis longispina*	6.73 ± 0.01 ^j^	1.49 ± 0.00 ^k^	1.88 ± 0.01 ^a^	70.51 ± 0.00 ^h^	1.94 ± 0.00 ^e^	30.91 ± 0.00 ^n^	144.81 ± 0.00 ^m^
*Englerophytum magalismontanum*	2.05 ± 0.00 ^a^	0.31 ± 0.00 ^d^	5.60 ± 0.00 ^h^	88.11 ± 0.00 ^o^	0.83 ± 0.00 ^b^	3.10 ± 0.00 ^b^	18.51 ± 0.00 ^a^
*Garcinia livingstonei*	5.85 ± 0.00 ^i^	0.69 ± 0.01 ^f^	4.80 ± 0.00 ^g^	81.59 ± 0.00 ^l^	2.16 ± 0.00 ^g^	4.64 ± 0.00 ^c^	33.41 ± 0.00 ^c^
*Halleria lucida*	5.06 ± 0.00 ^h^	1.44 ± 0.00 ^j^	10.13 ± 0.01 ^k^	64.98 ± 0.00 ^g^	6.98 ± 0.00 ^m^	11.41 ± 0.00 ^f^	86.52 ± 0.01 ^i^
*Manilkara mochisia*	3.65 ± 0.00 ^d^	1.38 ± 0.00 ^i^	12.77 ± 0.00 ^m^	43.03 ± 0.00 ^b^	2.19 ± 0.00 ^h^	36.98 ± 0.00 ^o^	169.10 ± 0.00 ^o^
*Parinari curatellifolia*	3.69 ± 0.00 ^de^	0.25 ± 0.00 ^c^	8.88 ± 0.00 ^j^	63.39 ± 0.01 ^f^	2.61 ± 0.00 ^i^	21.18 ± 0.00 ^k^	97.37 ± 0.01 ^j^
*Pappea capensis*	3.42 ± 0.02 ^c^	5.11 ± 0.00 ^m^	16.51 ± 0.00 ^n^	45.63 ± 0.00 ^c^	4.33 ± 0.01 ^l^	25.00 ± 0.00 ^m^	163.31 ± 0.00 ^n^
*Phoenix reclinata*	4.23 ± 0.23 ^g^	1.44 ± 0.00 ^j^	29.89 ± 0.00 ^o^	48.92 ± 0.00 ^d^	4.08 ± 0.01 ^k^	11.44 ± 0.00 ^g^	75.04 ± 0.01 ^g^
*Syzygium cordatum*	3.79 ± 0.00 ^ef^	0.34 ± 0.00 ^e^	7.64 ± 0.00 ^i^	81.78 ± 0.00 ^m^	0.83 ± 0.00 ^b^	5.62 ± 0.03 ^d^	28.86 ± 0.02 ^b^
*Syzygium guineense*	3.34 ± 0.00 ^c^	7.74 ± 001 ^n^	3.84 ± 0.00 ^e^	82.41 ± 0.00 ^n^	1.66 ± 0.00 ^d^	1.01 ± 0.00 ^a^	80.34 ± 0.00 ^h^
RDA (g/day)							
FDA		78	28	2.7–3.7 L	50	275	±2000 kcal/day
WHO	≤5.0 mg/100 g	44–77	18–35	2.1–2.6 L	28–65	130	1900–2900 kcal/day

Values are expressed as means ± standard deviations (*n* = 3). For all the values within a column, different letter superscripts mean significant differences (*p* < 0.05). Recommended daily allowance https://apps.who.int/iris/bitstream/handle/10665/43411/WHO_TRS_935_eng.pdf?ua=1 (accessed 20 January 2021).

**Table 4:** List of wild indigenous fruit plants, amino acids composition, and RDA values for indispensable amino acids.

Amino Acid	RDA (FDA)	RDA (WHO)	Source of Amino Acid (g/100 g)														
			Cm	Ce	Ca	Dc (EC)	Dc (NEL)	Dl	Em	Gl	Hl	Mm	Pc	Pcu	Pr	Sc	Sg
Arginine	N/A	N/A	0.35	0.30	0.29	0.39	0.50	3.31	0.39	0.47	0.55	0.30	0.51	0.25	0.67	0.52	0.38
Serine	N/A	N/A	0.20	0.22	0.28	0.24	0.33	1.14	0.32	0.32	0.64	0.21	0.46	0.44	0.31	0.23	0.36
Aspartic acid	N/A	N/A	0.43	3.34	0.47	0.45	0.66	0.52	0.80	0.62	0.62	0.34	0.75	0.51	0.81	0.55	0.55
Glutamic acid	N/A	N/A	0.48	0.51	0.64	0.57	0.75	0.62	0.71	0.74	0.85	0.45	1.10	1.63	1.07	0.58	0.62
Glycine	N/A	N/A	0.17	0.37	0.22	0.19	0.26	0.24	0.24	0.31	0.46	0.20	0.38	0.27	0.31	0.22	0.29
Threonine	20	15	0.17	0.16	0.22	0.20	0.28	0.23	0.21	0.26	0.30	0.17	0.31	0.25	0.27	0.19	0.23
Alanine	N/A	N/A	0.21	0.16	0.34	0.28	0.40	0.29	0.29	0.36	0.40	0.23	0.41	0.65	0.36	0.31	0.34
Tyrosine	33 *	25 *	0.05	0.10	0.14	0.04	0.04	0.16	0.38	0.47	0.27	0.11	0.22	0.18	0.19	0.14	0.19
Proline	N/A	N/A	0.15	0.18	0.24	0.19	0.28	0.25	0.26	0.28	0.27	0.19	0.34	0.24	0.23	0.19	0.21
HO-Proline	N/A	N/A	0.40	0.14	0.19	0.07	0.08	0.09	0.16	0.06	0.07	0.06	0.14	0.17	0.06	0.11	0.08
Methionine	33^	15^	0.07	0.05	0.05	0.04	0.08	0.10	0.09	0.08	0.08	0.05	0.15	0.05	0.11	0.06	0.07
Valine	24	39	0.17	0.14	0.29	0.22	0.31	0.27	0.31	0.37	0.39	0.23	0.37	0.32	0.37	0.25	0.30
Phenylalanine	33 *	25 *	0.14	0.12	0.21	0.18	0.24	0.22	0.22	0.28	0.31	0.15	0.30	0.22	0.27	0.20	0.24
Isoleucine	19	20	0.14	0.12	0.23	0.16	0.22	0.21	0.23	0.27	0.30	0.17	0.29	0.23	0.24	0.19	0.23
Leucine	42	39	0.23	0.18	0.31	0.27	0.36	0.36	0.35	0.46	0.47	0.28	0.48	0.34	0.42	0.32	0.39
Histidine	14	10	0.14	0.18	0.26	0.08	0.08	0.16	0.24	0.28	1.56	0.23	0.63	0.27	0.31	0.12	0.62
Lysine	38	30	0.30	0.26	0.53	0.27	0.28	0.55	0.54	0.67	0.58	0.37	0.77	0.46	0.58	0.49	0.61

RDA = Recommended dietary allowance (mg/kg/day) (https://www.who.int/nutrition/publications/nutrientrequirements/WHO_TRS_935/en/), * = phenylalanine + tyrosine, ^ methionine + cysteine, Cm = *Carissa macrocarpa*, Ce = *Carpobrotus edulis*, Co = *Cordyla africana*, Dc = *Dovyalis caffra*, Dl = *D. longispina*, Em = *Englerophytum magalismontanum*, Gl = *Garcinia livingstonei*, Hl = *Halleria lucida*, Mm =*Manilkara mochisia*, Pc = *Pappea capensis*, Pcu = *Parinari curatellifolia*, Pr = *Phoenix reclinata*, Sc = *Syzygium cordatum*, Sg = *S. guineense*.

## Data Availability

Not applicable.
